# The Claudin Family Protein FigA Mediates Ca^2+^ Homeostasis in Response to Extracellular Stimuli in *Aspergillus nidulans* and *Aspergillus fumigatus*

**DOI:** 10.3389/fmicb.2018.00977

**Published:** 2018-05-15

**Authors:** Hui Qian, Qiuyi Chen, Shizhu Zhang, Ling Lu

**Affiliations:** Jiangsu Key Laboratory for Microbes and Functional Genomics, Jiangsu Engineering and Technology Research Center for Microbiology, College of Life Sciences, Nanjing Normal University, Nanjing, China

**Keywords:** *Aspergillus*, calcium homeostasis, Ca^2+^ influx system, claudin protein, extracellular stimuli, ER stress

## Abstract

The claudin family protein Fig1 is a unique fungal protein that is involved in pheromone-induced calcium influx and membrane fusion during the mating of *Saccharomyces cerevisiae* and *Candida albicans*. Whether and how Fig1 regulates Ca^2+^ homeostasis in response to extracellular stimuli is poorly understood. Previously, we found *Aspergillus nidulans* FigA, a homolog of Fig1 in *S. cerevisiae*, similar to the high-affinity calcium uptake system, is required for normal growth under low-Ca^2+^ minimal medium. In this study, using the calcium-sensitive photoprotein aequorin to monitor cytosolic free calcium concentration ([Ca^2+^]_c_) in living cells, we found that the FigA dysfunction decreases the transient [Ca^2+^]_c_ induced by a high extracellular calcium stress. Furthermore, FigA acts synergistically with CchA (a high-affinity Ca^2+^ channel) to coordinate cytoplasmic Ca^2+^ influx in response to an extracellular Ca^2+^ stimulus. Moreover, FigA mediates ER stress-induced transient [Ca^2+^]_c_ in the presence or absence of extracellular calcium. Most importantly, these [Ca^2+^]_c_ responses mediated by FigA are closely related to its conserved claudin superfamily motif, which is also required for hyphal growth and asexual development in *A. nidulans*. Finally, the function of FigA in *Aspergillus fumigatus*, the most common airborne human fungal pathogen was studied. The result showed that the two FigA homologous in *A. nidulans* and *A. fumigatus* have a large degree of functional homology not only in asexual development but also in regulating transient [Ca^2+^]_c_. Our study expands the knowledge of claudin family protein FigA in Ca^2+^ homeostasis in response to extracellular stimuli.

## Introduction

Calcium is a highly versatile intracellular signal (second messenger) in eukaryotes. At resting state, the cytosolic Ca^2+^ concentration ([Ca^2+^]_c_) in fungal cells is very low, ranging from 50 to 100 nM (Cui et al., 2009; Liu S. et al., 2015). In response to various external stresses, a Ca^2+^-mediated signaling pathway is employed to regulate a wide variety of cellular processes through a transient increase in cytosolic Ca^2+^, which is elevated by activating the plasma membrane Ca^2+^ influx system and/or secreting Ca^2+^ from internal compartments (Berridge et al., 2000; Harren and Tudzynski, 2013; Munoz et al., 2015; Wang et al., 2016). In *Saccharomyces cerevisiae*, at least two different Ca^2+^ influx systems, the high-affinity Ca^2+^ influx system (HACS) and the low-affinity Ca^2+^ influx system (LACS) have been identified. The HACS was activated in response to mating pheromones, alkaline pH, oxidative stress and compounds such as azole-class antifungal agents or ER-stress agents (Muller et al., 2001; Popa et al., 2010; Zhang et al., 2016). The HACS is primarily composed of two subunits: the voltage-gated Ca^2+^ channel Cch1 (calcium channel) and the stretch-activated calcium channel/regulatory protein Mid1 (mating-induced death) (Iida et al., 1994; Fischer et al., 1997). Deletion of homologs of *mid1* and *cch1* results in calcium accumulation and growth defects under low-calcium conditions (Liu et al., 2006; Hallen and Trail, 2008; Wang et al., 2012; Harren and Tudzynski, 2013). Besides, Ecm7, as a new regulator of the HACS, directly or indirectly interacts with subunits of the HACS and may regulate the HACS through some unknown mechanisms in *S. cerevisiae* and *Candida albicans* (Martin et al., 2011; Ding et al., 2013).

**FIGURE 1:**
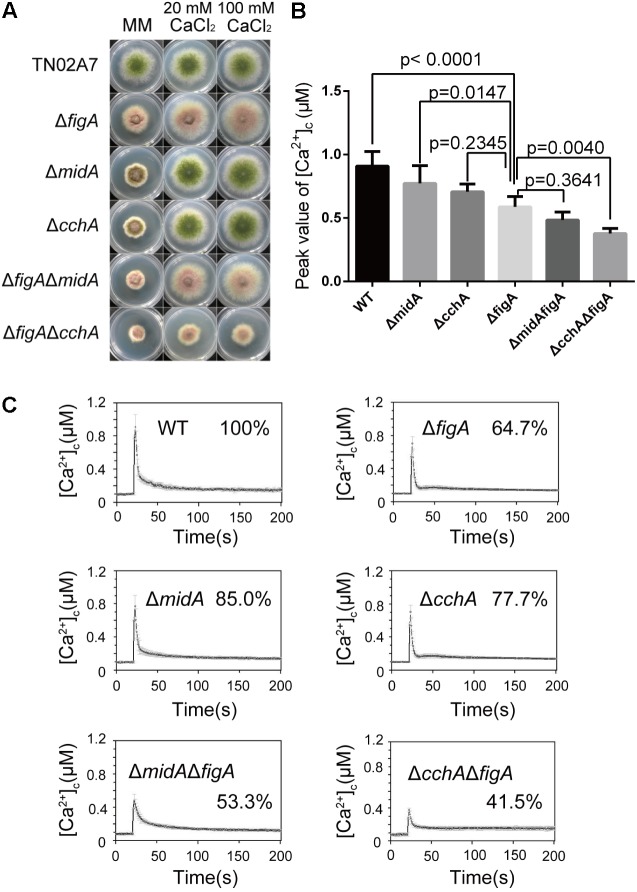
FigA and CchA act synergistically to coordinate cytoplasmic Ca^2+^ uptake. **(A)** Colony morphology comparison of the indicated strains grown on solid MM in the presence of 20 mM CaCl_2_, or 100 mM CaCl_2_ at 37°C for 2.5 days. **(B)** The bar graph shows the peak [Ca^2+^]_c_ of the indicated strains after treatment with CaCl_2_. Values represent averages of six independent experiments and error bars represent SD (*n* = 36). **(C)** Real-time monitoring of [Ca^2+^]_c_ of indicated strains following a stimulus of 0.1 M CaCl_2_ with the peak [Ca^2+^]_c_ amplitudes expressed as a percentage of that of the wild-type.

The HACS is responsible primarily for the calcium response in the low external Ca^2+^ concentration but suppressed under high external Ca^2+^ concentration, so that the LACS becomes essential for this response (Muller et al., 2001, 2003; Liu S. et al., 2015). Fig1 is the main component or regulator of LACS and was first identified as a pheromone-regulated protein involved in membrane fusion during yeast mating differentiation (Erdman et al., 1998). Fig1 was shown to be required for LACS activity, but not required for activation of Mpk1 mitogen-activated protein kinase in response to pheromones (Muller et al., 2001). LACS activity is insensitive to calcineurin activity, independent of Cch1p and Mid1p, and sufficient to elevate [Ca^2+^]_c_ in spite of its 16-fold lower affinity for Ca^2+^ (Muller et al., 2001). Consistent with the reports in *S. cerevisiae*, *Ca*Fig1 facilitates calcium influx during mating in *C. albicans* (Yang et al., 2011). *Ca*Fig1 is also involved in regulating the fungal hyphal thigmotropic orientation. However, deletion of *Cafig1* did not affect Ca^2+^ accumulation in low-Ca^2+^ conditions, suggesting the calcium independent roles of Fig1 (Brand et al., 2007). The function of Fig1 homologs has also been characterized in several filamentous fungi. In *Neurospora crassa*, loss of Fig1 decreased female fertility and arrested perithecium development in a mating type α background (Cavinder and Trail, 2012). In the plant pathogen *Fusarium graminearum*, loss of Fig1 resulted in phenotypes with reduced hyphal growth, failed perithecia and reduced virulence (Cavinder and Trail, 2012). Most recently, it was further showed that FigA in *F. graminearum* plays distinct roles from that of Mid1 and Cch1 in the formation of Ca^2+^ signature in hyphal cells (Kim et al., 2018).

Genus *Aspergillus* contains important species including the premier model filamentous fungus *Aspergillus nidulans* and the human pathogenic fungus *Aspergillus fumigatus*. The functions of Cch1 and Mid1 homologs (CchA and MidA) have been characterized in *A. nidulans*. Consistent with the function of Cch1 and Mid1 as a member of the HACS, CchA and MidA are required for conidial development, hyphal polarity establishment, and cell wall integrations in low-calcium environmental conditions (Wang et al., 2012). However, no Ecm7 homolog was reported in *Aspergillus.* Our previous work has demonstrated that FigA, the homolog of Fig1 in *A. nidulans*, is involved in hyphal growth, asexual and sexual development (Zhang et al., 2014). There are no clear homologs of Fig1 outside of fungi and it lacks the homology to any known ion influx channel. Instead, Fig1 shows similar secondary structure and topology to the claudin/PMP22/EMP superfamily. In mammals, claudin superfamily members are involved in vesicle trafficking and membrane-membrane interactions (Van Itallie and Anderson, 2006; Guenzel and Fromm, 2012; Overgaard et al., 2012). Although it has been proven that *S. cerevisiae* and *C. albicans* Fig1 is required for the low-affinity calcium transport during cell fusion (Muller et al., 2003; Yang et al., 2011). The roles of Fig1 and its homologs in the regulation of [Ca^2+^]_c_ in response to various external stimuli are not well-understood. In this study, by expressing a codon-optimized aequorin as a calcium probe in living hyphae of *A. nidulans* and *A. fumigatus*, we were able to monitor transient [Ca^2+^]_c_ in real-time. We found that FigA dysfunction significantly decreased the amplitude of the transient [Ca^2+^]_c_ induced by an extracellular high calcium stimulus and ER-stress caused by tunicamycin. Moreover, the conserved claudin motif of FigA was found to be essential for fungal growth and cytoplasmic Ca^2+^ regulation in *A. nidulans*.

## Results

### FigA and CchA Act Synergistically to Coordinate Cytoplasmic Ca^2+^ Influx in Response to an Extracellular Ca^2+^ Stimulus

Our previous data has shown that loss of CchA/MidA or FigA caused a reduced hyphal growth under calcium-limited minimal medium (MM) in *A. nidulans.* In contrast with Δ*cchA* and Δ*midA* mutants, there were no obvious differences in polarized growth and the sensitivity to the cell wall stressor Congo Red between the Δ*figA* mutant and wild-type in MM (Wang et al., 2012; Zhang et al., 2014) (**Supplementary Figure S1**). To further characterize the relationship between *figA* and *midA*/*cchA* in mediating calcium uptake, Δ*figA*Δ*midA* and Δ*figA*Δ*cchA* double deletion mutants were grown on MM and the hyphal radial growth were observed. The phenotypic defect in hyphal radial growth was exacerbated in the Δ*figA*Δ*cchA* and Δ*figA*Δ*midA* double deletion mutants compared to their respective parental single mutants Δ*figA*,Δ*cchA*, or Δ*midA* (**Figure 1A** and **Supplementary Figure S2**), which suggesting both CchA/MidA and FigA are required for fungal growth under low calcium conditions. Interestingly, the addition of 20 mM calcium to MM restored the radial growth defects (but not conidiation) in the Δ*figA*Δ*midA* mutant but not in the Δ*figA*Δ*cchA* mutant, suggesting non-overlapping roles for MidA and CchA. FigA and CchA appear to act synergistically to coordinate calcium uptake under calcium-limited conditions. To further explore the roles of FigA in the regulation of [Ca^2+^]_c_, real-time monitoring of [Ca^2+^]_c_ in living hyphal cells was performed. The pre-stimulatory resting [Ca^2+^]_c_ in *A. nidulans* is typically between 0.05 and 0.1 μM (Berridge et al., 2000; Munoz et al., 2015). As expected, the basal [Ca^2+^]_c_ resting level was approximately 0.09 μM in these experiments. Upon application of a 0.1 M CaCl_2_ stimulus (high external calcium), the highest [Ca^2+^]_c_ in wild-type cells increased to approximately 0.9 μM. Interestingly, it was the Δ*cchA* mutant (*p* = 0.0062) but not the Δ*midA* mutant (*p* = 0.1241) that showed a significant reduction of the transient [Ca^2+^]_c_ compared to its wild-type strain (100%) (**Figures 1B,C**). Surprisingly, the transient [Ca^2+^]_c_ in the *ΔfigA* mutant showed a similar decrease as the *ΔcchA* mutant under the same stimulus. Moreover, the Δ*figA*Δ*cchA* mutant, but not the Δ*figA*Δ*midA* mutant exhibited a dramatic further reduction in transient [Ca^2+^]_c_ compared to the Δ*figA* mutant under the same stimulus (**Figures 1B,C**). Collectively, these data suggest that FigA and CchA synergistically coordinate cytoplasmic Ca^2+^ influx in response to an extracellular calcium stimulus.

### FigA Is Required for [Ca^2+^]_c_ Transient in Response to the ER Stress

**FIGURE 2:**
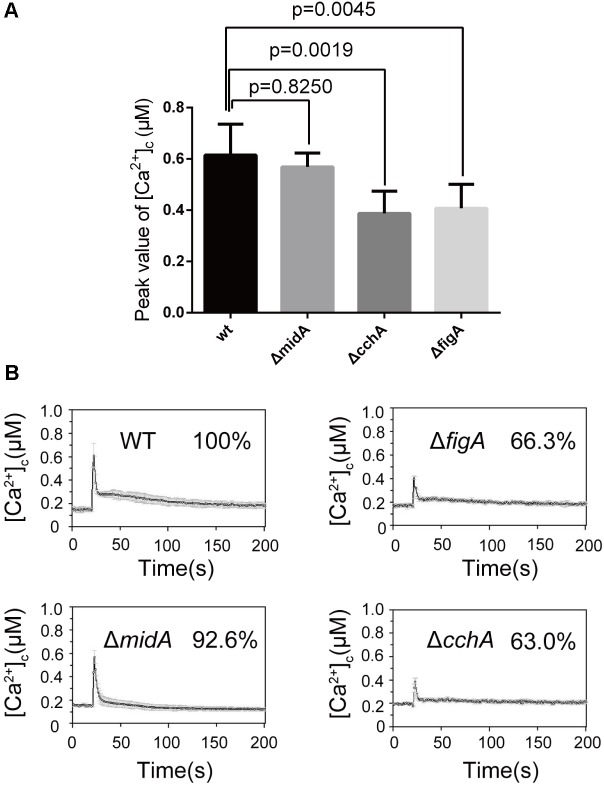
FigA regulates the transient [Ca^2+^]_c_ induced by ER stress following tunicamycin treatment. **(A)** The bar graph shows the peak [Ca^2+^]_c_ of the indicated strains after treatment with TM. Values represent averages of six independent experiments and error bars represent SD (*n* = 36). **(B)** Real-time monitoring of [Ca^2+^]_c_ of indicated strains to 5 μg/mL tunicamycin (TM) with the peak [Ca^2+^]_c_ amplitudes expressed as a percentage of that of the wild-type.

Endoplasmic reticulum (ER) is a multifunctional organelle required for calcium storage, protein folding and processing. Our previous data indicate that CchA, but not MidA influences ER stress-induced calcium influx in *A. nidulans* (Zhang et al., 2016). To explore the role of FigA in mediating transient [Ca^2+^]_c_ in response to the ER stress, we measured the transient [Ca^2+^]_c_ in hyphal cells following treatment with the ER-stress agent tunicamycin (TM). When the parental wild-type strain was treated with 5 μg/mL TM, an immediate transient increase in [Ca^2+^]_c_ to 0.6 μM was observed. Consistent with our previous result, the transient [Ca^2+^]_c_ in the Δ*cchA* mutant but not in Δ*midA* mutant showed a significant 27% reduction compared to the parental wild-type strain in response to TM (**Figures 2A,B**). Notably, the transient [Ca^2+^]_c_ in the Δ*figA* mutant decreased by approximately 24% compared to the parental wild-type strain under the same stimulus (**Figures 2A,B**). The results suggest that both CchA and FigA are involved in mediating the ER stress-induced calcium influx in *A. nidulans*.

**FIGURE 3:**
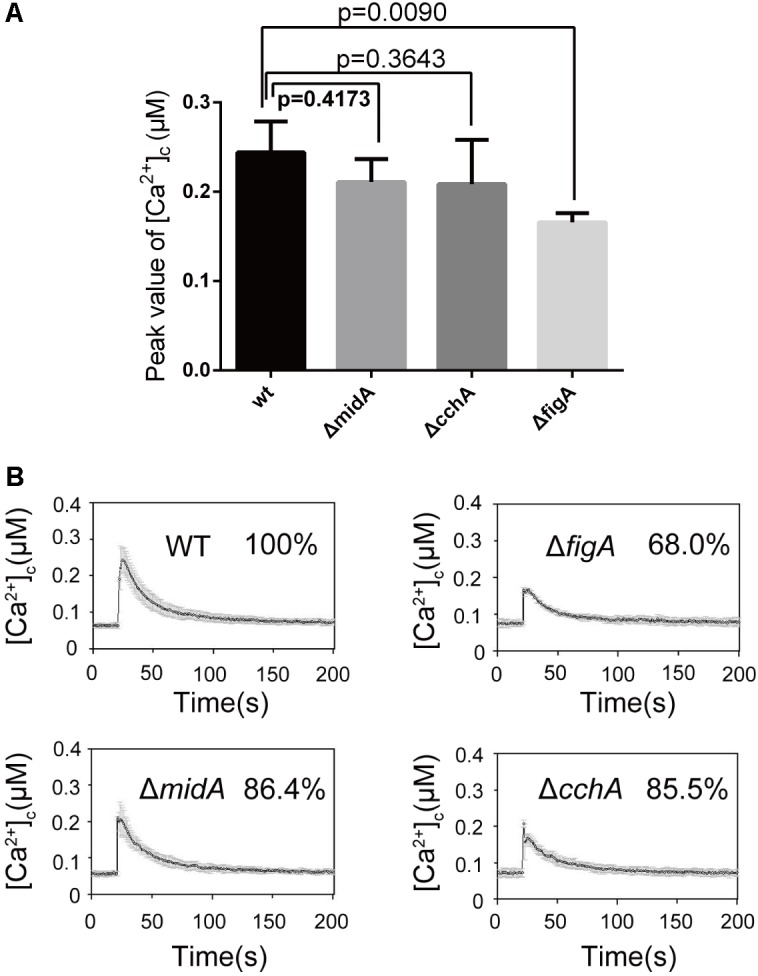
FigA contributes ER stress-induced transient [Ca^2+^]_c_ in the absence of extracellular calcium. **(A)** The bar graph shows the peak [Ca^2+^]_c_ of the indicated strains after treatment with EGTA and Tunicamycin (TM). Values represent averages of six independent experiments and error bars represent SD (*n* = 36). **(B)** Real-time monitoring of [Ca^2+^]_c_ of indicated strains to 5 μg/mL tunicamycin (TM) pretreated for 10 min with the calcium chelator EGTA (1 mM).

Furthermore, we tested whether the transient [Ca^2+^]_c_ resulting from the TM treatment is dependent on external calcium or internal calcium stores. Exposure of the Δ*figA* mutant cells to the calcium chelator EGTA (1 mM) prior to the TM treatment showed a significant 22% reduction in the transient [Ca^2+^]_c_ compared to wild-type. However, there was no significant difference between Δ*midA* or Δ*cchA* and the wild-type strain (**Figures 3A,B**). Collectively, these data showed that both CchA and FigA mediate ER stress-induced calcium influx in *A. nidulans*. Moreover, FigA also contributes to ER stress-induced transient [Ca^2+^]_c_ in the absence of extracellular calcium.

### The Claudin Motif Is Essential for FigA Function

**FIGURE 4:**
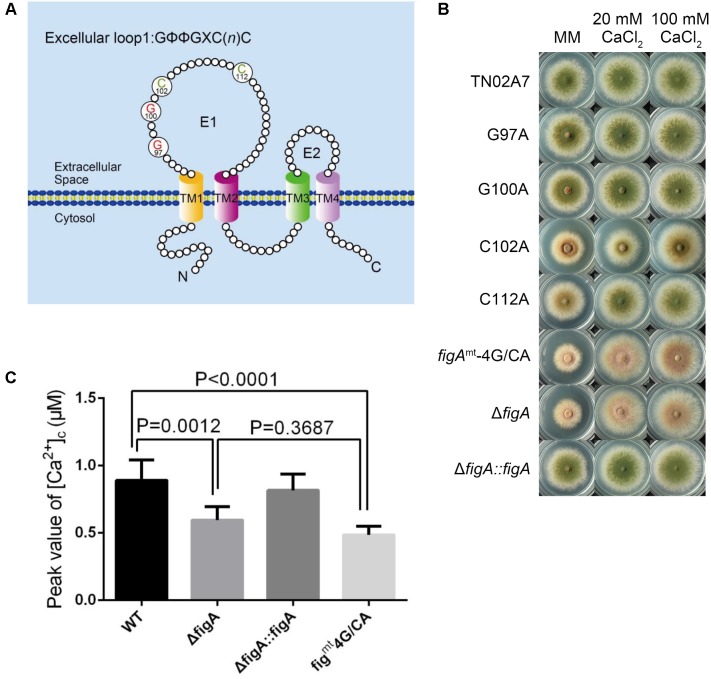
The Claudin motif of FigA is essential for hyphal growth, conidiation, and the regulation of transient [Ca^2+^]_c_ in *Aspergillus nidulans*. **(A)** Schematic diagram of conserved motifs of FigA in *A. nidulans* including the presence of four putative transmembrane domains with cytoplasmic N and C termini, two extracellular loops, and one short intracellular loop, and a conserved claudin motif [GΦΦGxC(n)C]. TM, transmembrane domain; E, excellular loop. **(B)** Colony morphology comparison of the indicated strains grown on solid MM in the presence of 20 mM CaCl_2_, or 100 mM CaCl_2_ at 37°C for 2.5 days. **(C)** The bar graph shows the peak [Ca^2+^]_c_ of the indicated strains following a stimulus of 0.1 M CaCl_2_. Values represent averages of six independent experiments and error bars represent SD (*n* = 36).

The conserved motif in FigA, GΦΦGxC(n)C, is a feature of the claudin superfamily. The glycine and cysteine residues in the conserved motif are located near the end of the first predicted extracellular loop region, between TM1 and TM2 (Yang et al., 2011; Zhang et al., 2014) (**Figure 4A**). Our previous studies have demonstrated that FigA is essential for hyphal growth, asexual, and sexual development in *A. nidulans* (Zhang et al., 2014). However, the molecular characteristics of the key FigA motifs have not yet been identified. Accordingly, we constructed individual site-directed FigA mutants at two conserved glycine residues (G97A and G100A), two cysteine residues (C102A and C112A), and a combined mutant at all four residues (*figA*^mt^-4G/CA). Plate assays revealed that G97A and G100A mutants displayed a wild-type like phenotype in hyphal growth and asexual conidiation either on MM or MM plus calcium media (**Figure 4B**). The C112A mutant displayed reduced conidia production on MM while exogenous calcium addition was able to almost completely restore the asexual conidiation defect in the C112A mutant. In comparison, the C102A mutant exhibited both hyphal and conidiation defects while the addition of calcium could completely restore the hyphal growth defect but only partially restore the conidiation defect in the C102A mutant. The *figA*^mt^-4G/CA mutant showed identical defects to a Δ*figA* mutant both in reduced colony size and lack of asexual conidia on MM. In addition, calcium supplementation completely rescued the hyphal growth retardation but did not restore the asexual conidiation defect in the *figA*^mt^-4G/CA and Δ*figA* mutants (**Figure 4B**). Since the above data showed that FigA plays an important role in transient [Ca^2+^]_c_ in response to external Ca^2+^, we determined the role of the conserved claudin motif of FigA on transient [Ca^2+^]_c_. As is shown in **Figure 4C**, the [Ca^2+^]_c_ in the *figA*^mt^-4G/CA point mutation strain decreased significantly to 55% compared with that in the wild-type, suggesting a role for the claudin motif on [Ca^2+^]_c_ regulation. In conclusion, we demonstrated that the conserved claudin motif is required for the proper function of FigA, including normal hyphal growth, conidiation, and the regulation of transient [Ca^2+^]_c_.

### FigA Homologs Display Functional Homology in *A. fumigatus*

**FIGURE 5:**
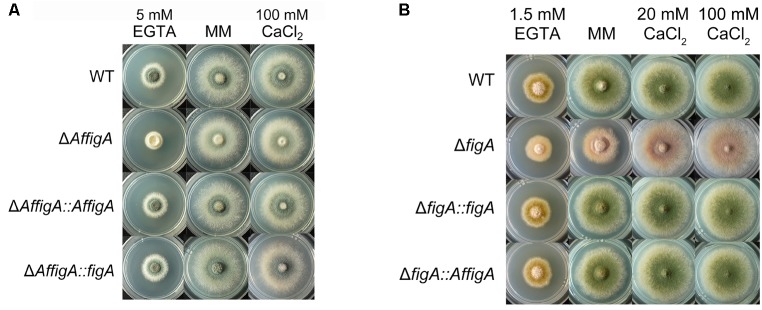
5 Plate assay. **(A)** Colony morphology comparison of the indicated *A. fumigatus* strains grown on solid MM in the presence of 5 mM EGTA or 100 mM CaCl_2_ at 37°C for 2.5 days. **(B)** Colony morphology comparison of the indicated *A. nidulans* strains grown on solid MM in the presence of 20 mM CaCl_2_, 100 mM CaCl_2_ or 1.5 mM EGTA.

*Aspergillus fumigatus* is the most common airborne fungal pathogen for human (Dagenais and Keller, 2009). FigA in *A. fumigatus* (KEGG Accession No. Afu3g09060) has 82% identity with its homolog *in A. nidulans*. Both proteins have conserved topology and contain the claudin motif. To explore the function of the FigA in *A. fumigatus*, an *AffigA* deletion strain was constructed by homologous gene replacement employing the *N. crassa pyr4* gene as a selectable marker (**Supplementary Figure S3A**). The resulting strain, Δ*AffigA*, was confirmed by diagnostic PCR and Southern blot (**Supplementary Figures S3B,C**). As is shown in **Figure 5A**, the loss of *figA* in *A. fumigatus* caused reduced conidia production on MM. Consistent with our observations in *A. nidulans*, the conidiation defects in the Δ*AffigA* mutant could not be rescued by the addition of extracellular calcium (**Figure 5A**). However, it seems that FigA plays a more important role in conidiation in *A. nidulans* than that in *A. fumigatus*. Conidia production in the Δ*AffigA* mutant was 20% of the reference strain (100%). In comparison, conidiation was almost completely abolished in the *A. nidulans figA* deletion strain. In addition, loss of *figA* in *A. fumigatus* did not affect the hyphal radial and polarized growth (**Figure 5A** and **Supplementary Figure S4**).

To verify the functional homology between the two FigA homologs in *A. nidulans* and *A. fumigatus*, two wild-type *figA* genes were used to cross complement each *figA* deletion strain. Functional assays showed that the *A. fumigatus* FigA could completely restore the asexual conidiation and hyphal growth defects seen in the *A. nidulans figA* deletion strain and vice versa under all test conditions including MM, MM plus EGTA or MM plus calcium (**Figures 5A,B**). The results indicated that the two FigA homologs in *A. fumigatus* and *A. nidulans* have a large degree of functional homology.

### FigA Mediates Transient [Ca^2+^]_c_ in *A. fumigatus*

**FIGURE 6:**
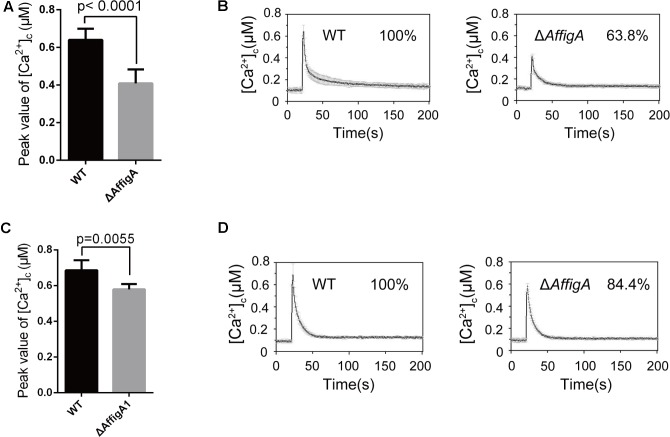
FigA mediates the transient [Ca^2+^]_c_ in *A. fumigatus*. **(A)** The bar graph shows the peak [Ca^2+^]_c_ of the indicated strains after treatment with CaCl_2_. Values represent averages of six independent experiments and error bars represent SD (*n* = 36). **(B)** Real-time monitoring of [Ca^2+^]_c_ of indicated strains following a stimulus of 0.1 M CaCl_2_ with the peak [Ca^2+^]_c_ amplitudes expressed as a percentage of that of the wild-type. **(C)** The bar graph shows the peak [Ca^2+^]_c_ of the indicated strains after treatment with TM. Values represent averages of six independent experiments and error bars represent SD (*n* = 36). **(D)** Real-time monitoring of [Ca^2+^]_c_ of indicated strains to 5 μg/mL tunicamycin (TM) with the peak [Ca^2+^]_c_ amplitudes expressed as a percentage of that of the wild-type.

The above results suggested that loss of *figA* affects calcium influx in *A. nidulans*. In order to explore whether *A. fumigatus* FigA regulates calcium influx, we monitored the transient [Ca^2+^]_c_ in response to extracellular calcium and TM by expressing codon-optimized aequorin in living cells of *A. fumigatus* wild-type A1160 and the Δ*AffigA* mutant. As expected, the [Ca^2+^]_c_ in the Δ*AffigA* mutant exposed to the 0.1 M CaCl_2_ stimulus significantly decreased by 26% compared to that of the parental wild-type strain (**Figures 6A,B**). When treated with the ER stressor TM, the [Ca^2+^]_c_ in the Δ*AffigA* mutant significantly decreased by 16% compared to that of the parental wild-type strain (**Figures 6C,D**). In summary, the above data showed that FigA possess a conserved role in [Ca^2+^]_c_ regulation in *A. fumigatus*.

## Discussion

The Ca^2+^-mediated signaling pathway plays a crucial role in fungal growth and survival under various stresses. In yeast, the HACS components Cch1 and Mid1 are the primary channels involved in Ca^2+^ homeostasis under low-Ca^2+^ conditions. The function of LACS component or regulator Fig1 seems to be confined to the pheromone-induced calcium influx in the rich medium which the function of the HACS is inhibited by calcineurin (Muller et al., 2001; Yang et al., 2011). The results presented herein show that FigA, the homolog of Fig1 in *S. cerevisiae*, plays crucial roles in the regulation of transient cytoplasmic Ca^2+^ concentrations induced by extracellular stimuli in *A. nidulans* and *A. fumigatus*. Several lines of evidence have shown that FigA in *Aspergillus* plays more important roles than that in yeasts. (I) FigA is required for the calcium uptake under low-Ca^2+^ conditions. Loss of *figA* causes a similar phenotype for retardation of hyphal growth to that of *cchA* and *midA* deletion mutants under low calcium conditions. And that growth defects are able to be restored by the addition of exogenous calcium. (II) FigA and CchA act synergistically to coordinate calcium uptake under calcium-limited conditions since the exogenous calcium-based restoration is abolished in a *figA* and *cchA* double deletion mutant. (III) FigA is required for the transient [Ca^2+^]_c_ in response to an extracellular calcium stimulus. The transient [Ca^2+^]_c_ in the *ΔfigA* mutant showed a similar amplitude decrease as the *ΔcchA* mutant following treatment with a high extracellular calcium stress stimulus. Moreover, double deletion of *figA* and *cchA* further decreases transient [Ca^2+^]_c_ than that in *ΔcchA* or *ΔfigA* mutants under the same stimulating condition. (IV) FigA also regulates the transient [Ca^2+^]_c_ in response to the ER stressor TM. It has been reported that ER stress can stimulate transient [Ca^2+^]_c_ through the HACS to promote fungal cell survival (Locke et al., 2000; Hong et al., 2010; Zhang et al., 2016). Interestingly, we found that in addition to the HACS component CchA, FigA is also involved in ER stress-induced calcium influx. However, unlike CchA, which is mainly responsible for calcium uptake from the extracellular environment, FigA mediates the ER stress-induced transient [Ca^2+^]_c_ in the absence of extracellular calcium, suggesting an important role of FigA in mediating the release of intracellular calcium stores under ER stress. Overall, the phenotypic assay combined with real-time monitoring of transient [Ca^2+^]_c_ provides strong evidences that FigA is required for fungal survival under low calcium conditions and the transient [Ca^2+^]_c_ in response to extracellular stimuli caused by extracellular calcium or ER stress.

This raises the question of how FigA regulates Ca^2+^ homeostasis. Unlike the MidA and CchA channels that all have EF hands, FigA is devoid of the Ca^2+^-binding motif. In addition, there is no FigA homolog in the known mammalian Ca^2+^ channels either. Therefore, it seems that FigA cannot bind calcium directly. FigA encodes a putative four-transmembrane domain protein (TM1, TM2, TM3, and TM4) that shares sequence similarity with members of the claudin superfamily. In mammals, the functions of claudin superfamily members are involved in epithelial tight-junction formation, which allow ions and other solutes to pass between cells (Van Itallie and Anderson, 2006; Guenzel and Fromm, 2012; Overgaard et al., 2012). The site-directed mutation experiments showed that the glycine and cysteine residues in the conserved motif [GΦΦGxC(n)C] of the claudin superfamily is important both for fungal low calcium adaptation and transient [Ca^2+^]_c_ in response to extracellular stimuli. In *S. cerevisiae*, Fig1 is localized predominantly to plasma membrane of shmoos and deletion of *ScFig1* results in incomplete fusions between tips of mating shmoos, which might be due to loss of a calcium-dependent membrane repair. However, such fusion defects were not observed in *fig1* null mutants of *C. albicans*. Instead, *Cafig1* presumably has an ability in maintaining the membrane stability during morphological transitions (Muller et al., 2003; Aguilar et al., 2007; Yang et al., 2011). However, FigA is located at the center of the septa of mature hyphae (probably around the pore) in *Aspergillus* (Zhang et al., 2014). The specific location of FigA at the center of hyphal septa indicates that FigA may also selectively allow solutes (calcium, for instance) to flow between cells (Harris, 2001; Zhong et al., 2012). Otherwise, FigA may work as a regulator of calcium channels.

Further studies will be required to identify and characterize the potential partners that interact with FigA. Overall, our study expands the knowledge of claudin family protein FigA in calcium homeostasis in response to extracellular stimuli.

## Materials and Methods

### Strains, Culture Condition, and Transformation

All fungal strains used in this study are listed in **Supplementary Table S1**. TN02A7, a strain with a deletion in the gene required for non-homologous end joining in double-strand break repair was used in transformation experiments as a parental wild-type strain of *A. nidulans* (Nayak et al., 2006). A1160, a parental wild-type strain of *A. fumigatus* was purchased from FGSC (Fungal Genetics Stock Center). All fungal strains were routinely cultured on MM with supplements to support the growth of relevant auxotrophic strains, as described previously (Zhang et al., 2014, 2016). Standard transformation procedures were performed according to a previously described method for *A. nidulans* and *A. fumigatus* (Osmani et al., 1988; May, 1989; Jiang et al., 2014).

### Plate Assays

To assess the influence of extracellular calcium on fungal growth, MM was supplemented with 20 mM CaCl_2_, 100 mM CaCl_2_, or 1.5 mM EGTA. 2 μl of 1 × 10^7^/ml *A. nidulans* conidia were spotted onto the relevant media. To assess the influence of extracellular calcium on *A. fumigatus*, MM was supplemented with 100 mM CaCl_2_ or 5 mM EGTA. 2 μl aliquots of 1 × 10^8^/ml *A. fumigatus* conidia from the indicated strains were spotted onto the relevant media. For the Congo Red sensitivity test, 2 μL aliquots (1 × 10^5^ conidia/mL, 1 × 10^6^ conidia/mL, 1 × 10^7^ conidia/mL, respectively) of indicated strains were spotted onto MM and MM plus 0.75 μg/mL Congo Red. All strains were cultured at 37°C for 2.5 days and then the colonies were observed and imaged.

### Construction of *A. nidulans* FigA Point Mutation Strains

Using genomic DNA (gDNA) of *A. nidulans* wild-type as the template, a 2.7 kb *figA* DNA fragment including a 1 kb upstream promoter region, a 0.9 kb coding sequence, and a 3′ flanking sequence was amplified with the primer pair FigAF/FigAR. The resulting fragment was digested with *Sma*I and *Pst*I and was cloned into the *Sma*I and *Pst*I digested plasmid pQa-*pyroA* to generate plasmid pFigA*-pyroA*. The Mut Express II Fast Mutagenesis kit (Vazyme^TM^) was used to construct plasmids carrying site-directed mutations. In brief, using the resulting plasmid pAnFigA*-pyroA* as a template, a DNA fragment with the complete ORF including the site-directed mutation (glycine^97^-alanine mutation), promoter sequence and 3′ UTR was amplified with the primers G97AF/G97AR. A similar strategy was used to construct plasmids carrying other mutations, including glycine^100^-alanine, cysteine^102^-alanine, cysteine^112^-alanine and the combined four mutated sites. All recombinant plasmids were individually transformed into the Δ*figA* mutant strain to generate relevant FigA point mutants.

### Constructions of *A. fumigatus figA* Deletion and Aequorin-Expressing Strains

To construct the *AffigA* deletion cassette in *A. fumigatus*, a fusion PCR based method was used as described previously (Szewczyk et al., 2006). In brief, 5′ and 3′ flanking DNA fragments of *AffigA* were amplified using the primers *AffigA*-P1/*AffigA*-P3, *AffigA*-P4 /*AffigA*-P6 from *A. fumigatus* wild- type A1160 gDNA. As a selectable marker, a 2.1 kb DNA fragment of *N. crassa pyr4* was amplified from the plasmid pAL5 using the primers Diag-*pyr4*-5′/Diag-*pyr4*-3′. The three PCR products were fused using primers *AffigA*-P2/*AffigA*-P5. The final PCR product was transformed into wild-type A1160 cells to construct the *figA* knockout strain. A diagnostic PCR was performed to ensure *figA* had been replaced by *pyr-4* at the original *figA* locus, using primers *AffigA*-P1/Diag-*pyr4*-3′.

For construction of the aequorin-expressing strains, the plasmid pAEQS1-15 containing a codon-optimized aequorin (Nelson et al., 2004) and the selective markers *riboB* or *hygB* were co-transformed into the indicated mutants. Transformants were screened for aequorin expression using methods described previously (Osmani et al., 1988; Denis and Cyert, 2002) and a high aequorin expressing strain was selected after homokaryon purification involving repeated plating of single conidia. All primers used to design constructs are listed in **Supplementary Table S2**.

### Southern Blot

To perform Southern blotting, the genomic DNA from the wild-type and the Δ*AffigA* strain were digested with *BamH*I/*EcoR*V and *BamH*I/*Sal*I respectively, separated by electrophoresis and transferred to a nylon membrane (Zeta-probe+; Bio-Rad). The fragment amplified with primers *AffigA* – southern – F and *AffigA* – southern – R was used as a probe to detect the Δ*AffigA* and wild-type strains, respectively. Labeling and visualization were performed using a digoxigenin (DIG) DNA labeling and detection kit according to the manufacturer’s instructions (Roche Applied Science, Indianapolis, IN, United States).

### Cross Complementation Assays for FigA Homologs

The *figA* genes from *A. fumigatus* and *A. nidulans* were cloned into pAN7-1 vector, which contained a hygromycin selection marker. Using gDNA of *A. fumigatus* wild-type A1160 as a template, a 2796 bp DNA fragment was amplified with the primer pair *Af*FigA-recon-P1/*Af*FigA-recon-P3, and then the fragment was cloned into plasmid pAN7-1 using the ClonExpress II OneStep Cloning Kit (Vazyme^TM^) to generate the plasmid pAfFigA-*hygB*. A similar strategy was used to clone *A. nidulans figA* into plasmid pAN7-1 to generate pFigA-*hygB* plasmid using the primer pair FigA-recon-P1/FigA-recon-P3 with gDNA of *A. nidulans* TN02A7 as a template.

To clone *A. fumigatus figA* into the pQa-*pyroA* vector, a 2.7 kb DNA fragment was amplified with the primer pair *Af*FigAF/*Af*FigAR using *A. fumigatus* A1160 gDNA as a template. The fragment was digested with *Sma*I and *Pst*I and ligated into *Sma*I and *Pst*I digested pQa-*pyroA* to generate the recombinant plasmid p*Af*FigA-*pyroA*. The plasmids p*Af*FigA-*hygB* and pFigA-*hygB* were transformed into the Δ*AffigA* mutant, and the plasmids p*Af*FigA-*pyroA* and pFigA*-pyroA* were transformed into Δ*figA* mutant to generate the relevant complemented strains.

### Intracellular [Ca^2+^]_c_ Measurement

Strains expressing the codon-optimized aequorin gene were grown on MM for 2.5 days to achieve maximal conidiation. 1 × 10^6^ spores in liquid media were dispensed in each well of a 96-well microtiter plate (Thermo Fischer, United Kingdom). Each experiment was performed as six replicates in the same multi well plate. Aequorin was reconstituted by incubating mycelia in 100 μl PGM containing 2.5 μM coelenterazine native (Biosynth AG, Rietlistrasse, Switzerland) for 4 h at 4°C in the dark. After reconstitution, mycelia were washed with two 1 ml washes in PGM and allowed to recover to room temperature for 1 h (Greene et al., 2002; Liu F.F. et al., 2015). To chelate extracellular Ca^2+^, 1 mM EGTA was added to each well 10 min prior to stimulus injection. At the end of each experiment, active aequorin was completely discharged by permeabilizing the cells with 20% (vol/vol) ethanol in the presence of excess Ca^2+^ (3 M CaCl_2_) to determine the total aequorin luminescence of each culture. Luminescence was measured with an LB 96P Microlumat Luminometer (Berthold Technologies, Germany), which was controlled by a dedicated computer running the Microsoft Windows-based Berthold WinGlow software. Relative light unit (RLU) values were converted into [Ca^2+^]_c_ concentrations using the following empirically derived calibration formula: pCa = 0.332588 (-logk) + 5.5593, where k is luminescence (in RLU) s^-1^/total luminescence (in RLU) (Nelson et al., 2004). Error bars represent the standard error of the mean from six independent experiments and percentages in the figures represent peak [Ca^2+^]_c_ compared to that of wild-type (100%).

### Statistical Analysis

Statistical differences were analyzed using GraphPad Prism 6 software (GraphPad Software). *p*-Values were calculated with one-way ANOVA for multiple comparisons and adjusted with Tukey correction and non-paired Student’s *t*-test where two groups were compared.

## Author Contributions

HQ, SZ, and LL: conception and design of the investigation and work. HQ and QC: completion of the experiments. HQ and SZ: evaluation and analysis of the results and manuscript writing. HQ, QC, SZ, and LL: final approval of manuscript.

## Conflict of Interest Statement

The authors declare that the research was conducted in the absence of any commercial or financial relationships that could be construed as a potential conflict of interest.

## Supplementary Material

FIGURE S1 Congo Red sensitivity test. 2 μL aliquots (1 × 10^5^ conidia/mL, 1 × 10^6^ conidia/mL, 1 × 10^7^ conidia/mL, respectively) of indicated strains were spotted onto MM and MM plus 0.75 μg/mL Congo Red and cultured for 2.5 days at 37°C.

FIGURE S2 Quantitative data of the colony diameter of the indicated strains. The control strain (TN02A7) and the Δ*figA*, Δ*midA*, Δ*figA*Δ*midA*, Δ*cchA*, Δ*figA*Δ*cchA* mutants were grown on MM at 37°C for 2.5 days and then the hyphal radial growth was quantified. Error bars represent standard deviations from three replicates.

FIGURE S3 Constructions of *AffigA* deletion strain. **(A)** Diagrams showing the strategy for generating *AffigA* deletion strain. The *AffigA* gene was replaced with the *pyr4* expression cassette to create the Δ*AffigA* mutant. **(B)** Diagnostic PCR was employed to verify the mutant. For lanes 1 and 2, the PCR primers Diag-del-*AffigA*-5′/Diag-del-*AffigA*-3′ were used to detect whether *AffigA* existed in the genome. For lanes 3 and 5, the PCR primers *AffigA*-P1/Diag-*pyr4*-3′ (lane 3) and Diag-*pyr4*-5′/*AffigA*-P6 (lane 5) were used, respectively, to verify homologs replacement of *AffigA* by *pyr4* marker. For lanes 2, 4 and 6, genomic DNA of parental strain was used as PCR template; for lanes 1, 3 and 5, the template was genomic DNA of Δ*AffigA* mutant. **(C)** Southern blot. The DIG-labeled probe bound to a 1072 and 2214 bp fragment in the wild-type and Δ*AffigA* strains respectively, indicating the replacement of *AffigA* by *pyr4*.

FIGURE S4 The morphologic observation of hyphae. The micrographs of Different Interference Constrast (DIC) of indicated strains. There was no significant difference in hyphal polarity between the Δ*AffigA* strain and wild-type (A1160). Bars: 10 μm.

TABLE S1 All fungal strains used in this study.

TABLE S2 Primers used in this study.
