# Supplementary figures and images for: The Claudin Family Protein FigA Mediates Ca2+ Homeostasis in Response to Extracellular Stimuli in Aspergillus nidulans and Aspergillus fumigatus
Source: Front Microbiol. 2018 May 15;9:977. doi: 10.3389/fmicb.2018.00977 (PMC5962676; doi:10.3389/fmicb.2018.00977)

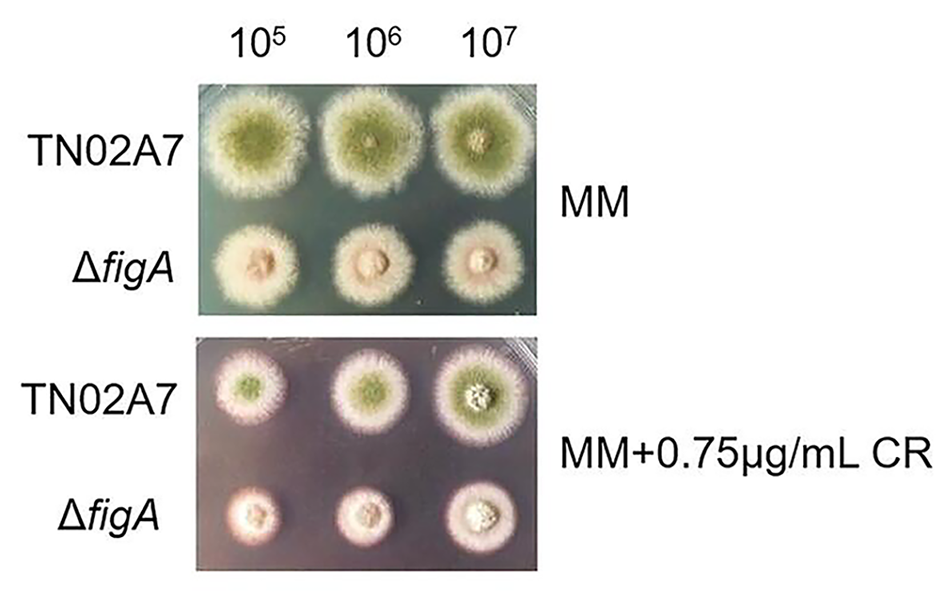

Supplement: FIGURE S1 — Congo Red sensitivity test. 2 μL aliquots (1 × 10^5^ conidia/mL, 1 × 10^6^ conidia/mL, 1 × 10^7^ conidia/mL, respectively) of indicated strains were spotted onto MM and MM plus 0.75 μg/mL Congo Red and cultured for 2.5 days at 37°C. [file Image_1.TIF]

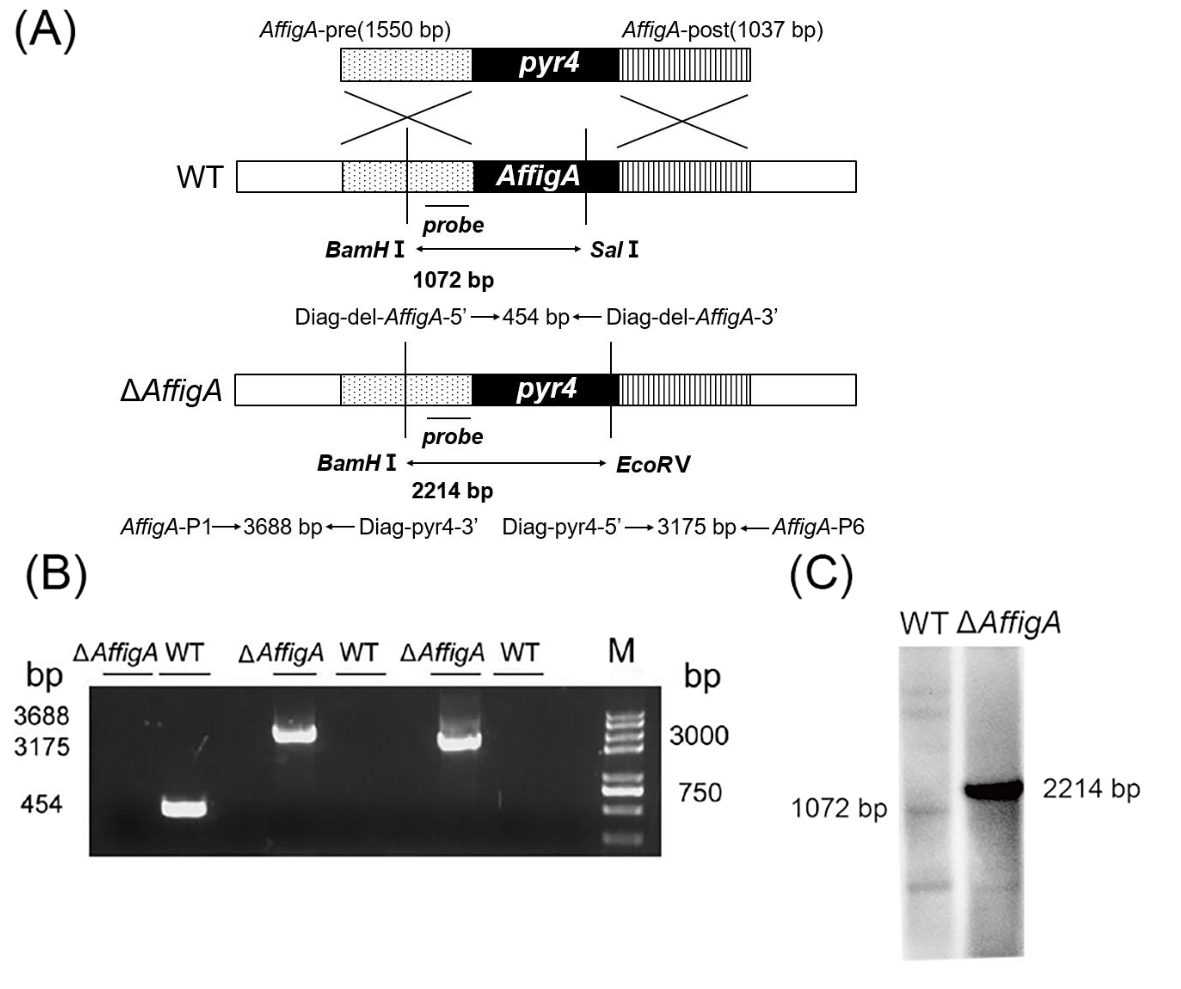

Supplement: FIGURE S3 — Constructions of AffigA deletion strain. (A) Diagrams showing the strategy for generating AffigA deletion strain. The AffigA gene was replaced with the pyr4 expression cassette to create the ΔAffigA mutant. (B) Diagnostic PCR was employed to verify the mutant. For lanes 1 and 2, the PCR primers Diag-del-AffigA-5′/Diag-del-AffigA-3′ were used to detect whether AffigA existed in the genome. For lanes 3 and 5, the PCR primers AffigA-P1/Diag-pyr4-3′ (lane 3) and Diag-pyr4-5′/AffigA-P6 (lane 5) were used, respectively, to verify homologs replacement of AffigA by pyr4 marker. For lanes 2, 4 and 6, genomic DNA of parental strain was used as PCR template; for lanes 1, 3 and 5, the template was genomic DNA of ΔAffigA mutant. (C) Southern blot. The DIG-labeled probe bound to a 1072 and 2214 bp fragment in the wild-type and ΔAffigA strains respectively, indicating the replacement of AffigA by pyr4. [file Image_3.TIF]

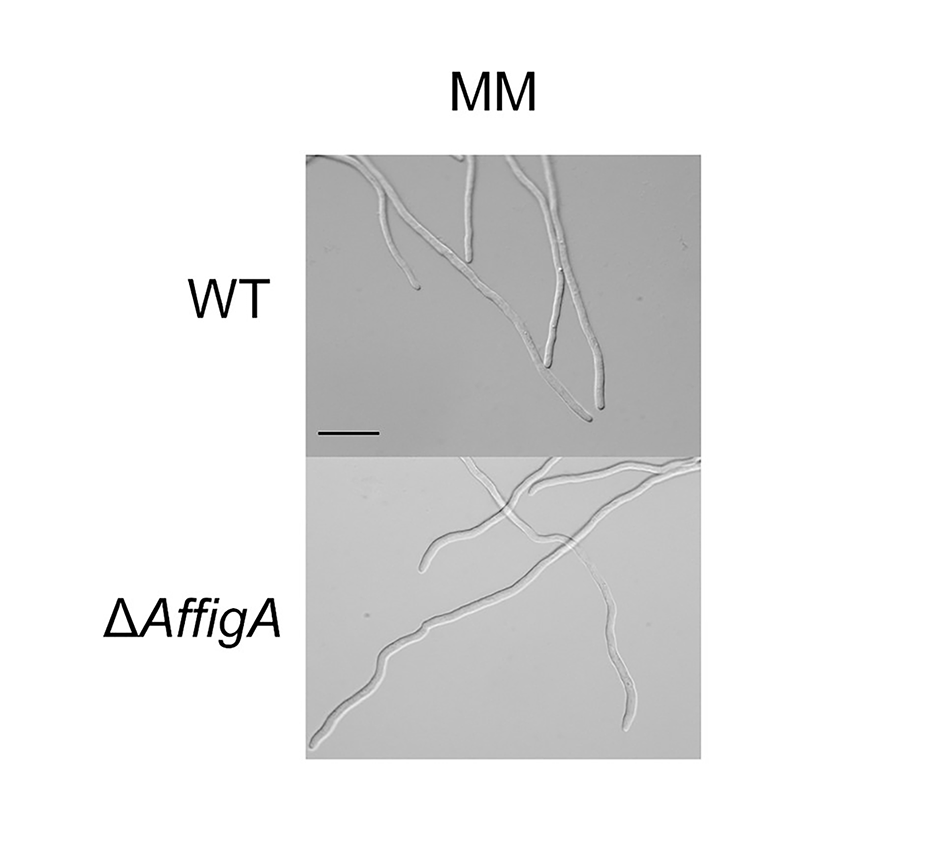

Supplement: FIGURE S4 — The morphologic observation of hyphae. The micrographs of Different Interference Constrast (DIC) of indicated strains. There was no significant difference in hyphal polarity between the ΔAffigA strain and wild-type (A1160). Bars: 10 μm. [file Image_4.TIF]
