# Supplementary material for: The Claudin Family Protein FigA Mediates Ca2+ Homeostasis in Response to Extracellular Stimuli in Aspergillus nidulans and Aspergillus fumigatus
Source: Front Microbiol. 2018 May 15;9:977. doi: 10.3389/fmicb.2018.00977 (PMC5962676; doi:10.3389/fmicb.2018.00977)
Supplement: TABLE S1 — All fungal strains used in this study. [file Table_1.docx]

**Supplemental Table 1**.

All fungal strains used in this study.

| **Strain Name** | **Gene deletion** | **Genotype** | **Source** |
| --- | --- | --- | --- |
| TN02A7 | wild type | *pyrG89;pyroA4,nkuA::argB2;riboB2* | FGSC |
| A1160 | wild type | Δ*aku80, pyrG1* | FGSC |
| QH01 | Δ*figA* | *pyrG89;pyroA4;nkuA::argB2;*Δ*ﬁgA::pyrG;riboB2,veA1* | ([Zhang et al., 2014](#_ENREF_2)) |
| QH02 | Δ*midA* | *pyrG89;*Δ*midA::pyrG,pyroA4, nKuA::argB2; riboB2* | ([Wang et al., 2012](#_ENREF_1)) |
| QH03 | Δ*cchA* | *pyrG89;*Δ*cchA::pyrG ,pyroA4,nkuA::argB2;riboB2* | ([Wang et al., 2012](#_ENREF_1)) |
| QH04 | Δ*ﬁgA*Δ*midA* | *pyrG89;*Δ*midA::pyroA;pyroA4,nkuA::argB2;*Δ*ﬁgA::pyrG;riboB2,veA1* | ([Zhang et al., 2014](#_ENREF_2)) |
| QH05 | Δ*ﬁgA*Δ*cchA* | *pyrG89;pyroA4,nkuA::argB2;* Δ*ﬁgA::pyrG;*Δ*cchA::pyrG;riboB2,veA1* | ([Zhang et al., 2014](#_ENREF_2)) |
| QH08 | TN02A7-AEQ | *pyrG89;nkuA::argB2;riboB2; pAEQ-aeqS* | ([Zhang et al., 2016](#_ENREF_3)) |
| QH09 | Δ*midA*-AEQ | Δ*midA::pyrG;pyrG89;nkuA::argB2;riboB2;pAEQ-aeqS* | ([Zhang et al., 2016](#_ENREF_3)) |
| QH10 | Δ*cchA*-AEQ | Δ*cchA::pyrG;pyrG89;nkuA::argB2;riboB2;pAEQ-aeqS* | ([Zhang et al., 2016](#_ENREF_3)) |
| QH11 | Δ*figA*-AEQ | *pyrG89;nkuA::argB2;*Δ*ﬁgA::pyrG;riboB2,veA1;pAEQ-aeqS* | This work |
| QH12 | Δ*ﬁgA*Δ*midA*-AEQ | *pyrG89;*Δ*midA::pyroA;pyroA4,nkuA::argB2;*Δ*ﬁgA::pyrG;veA1;pAEQ-aeqS* | This work |
| QH13 | Δ*ﬁgA*Δ*cchA*-AEQ | *pyrG89;pyroA4,nkuA::argB2;*Δ*ﬁgA::pyrG;*Δ*cchA::pyrG; veA1;pAEQ-aeqS* | This work |
| QH14 | G97A | *pyrG89;nkuA::argB2;*Δ*AnﬁgA::pyrG;AnfigA*^G97A^*;riboB2,veA1* | This work |
| QH15 | G100A | *pyrG89;nkuA::argB2;*Δ*AnﬁgA::pyrG;AnfigA^100A^;riboB2,veA1* | This work |
| QH16 | C102A | *pyrG89;nkuA::argB2;*Δ*AnﬁgA::pyrG;AnfigA^C102A^;riboB2,veA1* | This work |
| QH17 | C112A | *pyrG89;nkuA::argB2;*Δ*AnﬁgA::pyrG;AnfigA^C112A^;riboB2,veA1* | This work |
| QH18 | *figA^mt^*-4G/CA | *pyrG89;nkuA::argB2;*Δ*AnﬁgA::pyrG;AnfigA^G97A G100A C102A C112A^;riboB2,veA1* | This work |
| QH19 | Δ*figA::figA* | Δ*figA::pyrG;pyrG89;nkuA::argB2; figA::* *pyroA* | This work |
| QH20 | Δ*figA::figA*-AEQ | Δ*figA::pyrG;pyrG89;nkuA::argB2;* *figA:: pyroA;pAEQ-aeqS* | This work |
| QH21 | *figA*^mt^-4G/CA-AEQ | *pyrG89;nkuA::argB2;ΔAnﬁgA::pyrG;AnfigA^G97A G100A C102A C112A^;riboB2,veA1;pAEQ-aeqS* | This work |
| QH22 | Δ*figA::AffigA* | *pyrG89;nkuA::argB2;*Δ*ﬁgA::pyrG;riboB2,veA1;AffigA::hygB* | This work |
| QH23 | Δ*AffigA* | *Af1160,* Δ*AffigA::pyr4* | This work |
| QH24 | Δ*AffigA::AffigA* | *Af1160,*Δ*AffigA::pyr4,AffigA::hygB* | This work |
| QH25 | Δ*AffigA::figA* | *Af1160,*Δ*AffigA::pyr4,AnfigA::hygB* | This work |
| QH26 | *Af*1160-AEQ | Δ*aku80, pyrG1;pAEQ-aeqS, hygB* | This work |
| QH27 | Δ*AffigA*-AEQ | *Af1160,*Δ*AffigA::pyr4;pAEQ-aeqS, hygB* | This work |

**Supplemental Table 2**.

Primers used in this study.

| **Name** | **Sequence (5’ to 3’)** | **Purpose** |
| --- | --- | --- |
| FigAF | TCCCCCGGGTGTACATATTATTGGTTTATCAAAGC | Complementation assay |
| FigAR | AACTGCAGCAATTTGTGCATTCCCAGCTG |  |
| *Af*FigAF | TCCCCCGGGTCAACCTTGTCTGGTCCTTTCC |  |
| *Af*FigAR | AACTGCAGGTCCACTTTCCATTCGTGTCG |  |
| G97AF | CGCGTAGCATATTTTGGCATTTGTGTCCAACC | Site-directed mutagenesis |
| G97AR | CCAAAATATGCTACGCGAACCTCCAGCTGAGC |  |
| G100AF | TGCCATTTGTGTCCAACCAGACGGTGGATCTT |  |
| G100AR | GTTGGACACAAATGGCAAAATATCCTACGCGAACCTCC |  |
| C102AF | TTGGCATTGCTGTCCAACCAGACGGTGGATCTT |  |
| C102AR | GTTGGACAGCAATGCCAAAATATCCTACGCGAA |  |
| C112AF | CATCGCCAATAACAACGCAACCGCACTTGCAGA |  |
| C112AR | GCGTTGTTATTGGCGATGTAAGATCCACCGTCTGGTTG |  |
| *figA*^mt^-4G/CAF | TTGCTGTCCAACCAGACGGTGGATCTTACATCGCCAATAACAACGCAACCGCACTT |  |
| *figA*^mt^-4G/CAR | GTCTGGTTGGACAGCAATGGCAAAATATGCTACGCGAACCTCCAGCTGA |  |
| *AffigA*-P1 | AAAGTACATCTTCAAGCCCCAGTC | Fusion PCR for *AffigA* knockout |
| *AffigA*-P2 | TGGCCTAGAAAGTTTGGGAGG |  |
| *AffigA*-P3 | CGATTAAGTTGGGTAACGCCATGCGAGAGCAGATTCAGATCTC |  |
| *AffigA*-P4 | ATAAGTAGCCAGTTCCCGAAAGCCCATAATCCGCAGCACTCTTG |  |
| *AffigA*-P5 | AGCCCACATTTCTGTTCCCAC |  |
| *AffigA*-P6 | TTGGTTGTCGCATTACAGCAT |  |
| Diag-del-*AffigA*-5’ | CTTCCCATTTACTTCGTATTCCAG | *AffigA* knockout confirmation |
| Diag-del-*AffigA*-3’ | CGATGCCACCCAGATTAGATTC |  |
| Diag-*pyr4*-5’ | TGGCGTTACCCAACTTAATCG | Selection marker gene |
| Diag-*pyr4*-3’ | GCTTTCGGGAACTGGCTACTTAT |  |
| *Af*FigA-recon-P1 | GAGGTAATCCTTCTTTCTAGATCAACCTTGTCTGGTCCTTTCC | *Af*FigA complementation |
| *Af*FigA-recon-P3 | ACGACGGCCAGTGCCAAGCTTGTCCACTTTCCATTCGTGTCG |  |
| FigA-recon-P1 | GAGGTAATCCTTCTTTCTAGATGGAGTTTGGAAGAGGCTAACAG | FigA complementation |
| FigA-recon-P3 | ACGACGGCCAGTGCCAAGCTTGCTTATCTTACGCCTAATCCCAGT |  |
| *AffigA*-southern-F | TGTTCTTCCTAAACTATACCC | Southern blot |
| *AffigA*-southern-R | AGAGCAGATTCAGATCTCCC |  |
